# Automated Detection of Atypical Aviation Obstacles from UAV Images Using a YOLO Algorithm

**DOI:** 10.3390/s22176611

**Published:** 2022-09-01

**Authors:** Marta Lalak, Damian Wierzbicki

**Affiliations:** 1 Institute of Navigation, Polish Air Force University, 08-521 Dęblin, Poland; 2 Department of Imagery Intelligence, Faculty of Civil Engineering and Geodesy, Military University of Technology, 00-908 Warsaw, Poland

**Keywords:** detection, UAV, point cloud, classification, deep learning, YOLO, aviation obstacle

## Abstract

Unmanned Aerial Vehicles (UAVs) are able to guarantee very high spatial and temporal resolution and up-to-date information in order to ensure safety in the direct vicinity of the airport. The current dynamic growth of investment areas in large agglomerations, especially in the neighbourhood of airports, leads to the emergence of objects that may constitute a threat for air traffic. In order to ensure that the obtained spatial data are accurate, it is necessary to understand the detection of atypical aviation obstacles by means of their identification and classification. Quite often, a common feature of atypical aviation obstacles is their elongated shape and irregular cross-section. These factors pose a challenge for modern object detection techniques when the processes used to determine their height are automated. This paper analyses the possibilities for the automated detection of atypical aviation obstacles based on the YOLO algorithm and presents an analysis of the accuracy of the determination of their height based on data obtained from UAV.

## 1. Introduction

In recent years, a rapid development of investment areas in large cities has been witnessed. This involves intensive construction works of new objects. Such works often require the use of additional equipment, such as cranes, lifting cranes, etc., whose position and height are often of great importance, especially if the works are conducted in the direct vicinity of an airport. In such an event, these objects become temporary aviation obstacles. Accurate data indicating the location and height of this type of obstacles are necessary to ensure safety in the air space. Information about temporary aviation obstacles is provided in the NOTAM (Notice to Airmen) announcements and is delivered by the air traffic controller who supervises the security of the aviation operations. The emergence of an aviation obstacle of temporary nature is the main factor that may generate risk in the direct proximity of the airport. The area where the obstacle is located must be constantly monitored by the airport manager [1]. However, temporary aviation obstacles are not the only elements that pose a threat to air traffic safety. Permanent obstacles situated in the vicinity of an airport also carry a risk and require flight procedures that will take into account the elevation of such obstacles to be developed. There are certain recommendations concerning the methods of obtaining data about obstacles, where an emphasis is placed on the automation of processes for the purposes of collecting large sets of data. Still, although the requirements and techniques for obtaining data about aviation obstacles have been systematised, the automation of the mechanisms used to obtain data about elongated obstacles, such as antennas, masts, etc., is still being developed [1]. In order to capture very thin objects, a larger image scale than one used for traditional exploratory flights is required. This, in turn, requires lower altitudes. The techniques used so far for aviation obstacle detection have been based on the use of point clouds from airborne laser scanning (ALS). These techniques have several drawbacks. To begin with, missing an elongated obstacle is impossible [1]. Secondly, the object detection control is performed with the use of traditional ground measurements, which extend the entire process and eliminate the possibility of its automation. Furthermore, to ensure safety in the airspace, it is necessary to maintain the obstacle data, updating it regularly. Finally, the airborne laser scanning does not provide a high time resolution of data acquisition. According to the latest provisions of the Eurocontrol [1] manual, the detection of obstructions of an elongated shape should be provided at a much larger image scale than that obtained in the case of traditional photogrammetric flights. This is possible with a lower flight altitude obtained with UAV flights. At a lower flight altitude, the obtained spatial accuracy (X, Y, Z) will be the highest. Updating information about aviation obstacles involves obtaining and processing large amounts of data. As a result, the process of obtaining and processing these data must be automated. Currently, the process of reporting temporary aviation obstacles takes a long time and often requires traditional, time-consuming geodetic measurements. The automation of the process of obtaining data about temporary aviation obstacles and ensuring the required accuracy levels for coordinates X, Y, Z of the obstacle pose a new challenge in the research on obtaining data, which has been reliable so far.

The authors of this paper presented an innovative methodology for the automatic detection and classification of elongated aviation obstacles based on data obtained from unmanned aerial vehicles. The novelty of their approach consists of the fusion of the YOLOv3 algorithm operating with the use of neural networks extracting features from the image for the detection of atypical aviation obstacles. The authors also proposed and used a new algorithm for obstacle classification, which is based on a dense point cloud for the estimation of the height of these obstacles.

## 2. Related Works

Currently, aerial photogrammetry is the most efficient technique of collecting data about obstacles, although it involves less automation than other techniques, such as ALS (airborne laser scanning). The binding requirements for detecting atypical aviation obstacles, which usually are “thin” objects of an elongated shape, require lower altitude of flight than those applied in traditional aerial photogrammetry [1]. Achieving a lower flight altitude becomes possible thanks to the use of unmanned aerial vehicles (UAVs). At lower altitudes, the spatial accuracy (X, Y, Z) of temporary obstacles is much higher. Apart from the altitude of flight, the temporal resolution of obtaining data as well as the availability and low operating costs offer an attractive alternative for traditional teledetection platforms [2,3,4,5,6]. UAVs, which are used increasingly often, provide digital images that are used to create dense point clouds to describe 3D objects [7]. This creates new possibilities of object classification based on point clouds combined with the use of the properties that are present in the images from the data obtained by UAVs [8]. However, there is an emerging need to develop the automation of the processes in order to address the challenge of detecting atypical aviation obstacles of elongated shapes where the correct detection is only possible at such a low altitude of flight that can be ensured by a UAV.

### 2.1. Object Classification Based on RGB Imagery

Extracting objects from high-resolution images obtained by UAVs plays an important role in geospatial applications, including urban planning, telecommunications, disaster monitoring, navigation, updating geographic databases, and dynamic monitoring of cities. Automated extraction of objects is a challenging task, as the objects in various regions have different spectral and geometric properties. As a result, classic image processing techniques are insufficient for automated extraction of objects from high-resolution data. The deep learning and semantic segmentation models, which have become popular in recent years, are used to extract objects from high-resolution images in an automated way. However, the effective classification, detection, and segmentation of various objects in remote sensing images also poses a challenge for scientists due to various factors, such as the appearance of the object, various backgrounds, and environmental conditions. In general, image segmentation is a process that enables labelling pixels in the input image, so that the pixels in the same region/area or object are correlated with the same class label. It helps to determine whether the given UAV image contains one or more objects that belong to the category of interest and locate any predicted position of the object in the image.

Object detection based on deep learning may be divided into two categories: two-stage and single-stage detection. The RCNN (Region Based Convolutional Neural Networks) (RCNN [9], Fast RCNN [10], and Faster RCNN [11]) series are a two-step algorithm, whose accuracy highly exceeds that of many other detection algorithms. However, this type of approach requires higher computational costs, which extends the processing time. In the single-stage category, one may distinguish the SSD (Single Shot MultiBox Detector) [12,13] and the YOLO (You Only Look Once) algorithm proposed by Joseph Redmon and Ross Girshick [14]. YOLO solves the detection of objects like a regression problem and displays the position and classification of the object in an end-to-end network within a single step. Due to the detection speed, it is currently one of the most commonly used algorithms. The YOLO algorithm is being constantly improved due to the significant errors emerging in the accuracy of the detection of small objects. The version that deserves attention is YOLOv3, which applies the method of grouping K-averages in order to automatically select the best initial regression frame for the dataset. The multi-scale anchor mechanism [15] is adapted to improve the accuracy of detecting small objects.

Anguelov et al., presented in the work by Liu, D., proposed an SSD algorithm that uses the regression method for detection, integrates the positioning, and classifies it within a single network. The SSD was modified into VGG16 [16], to replace the fully integrated VGG16 layer with a convolutional layer.

As opposed to other methods, the deep learning methods are capable of distinguishing the low and high-level properties automatically [17,18]. Such deep learning methods as the Convolutional Neural Networks (CNNs) use convolutions to distinguish features automatically. In 2014, pixel-based classification was enabled as a result of adapting the CNNs model to a fully convolutional neural network [19]. Since then, deep learning methods have often been used in research on semantic segmentation and object extraction [20]. In recent years, research with the use of deep learning methods has been conducted in various areas of remote sensing, including pre-processing of images [21], detecting objects [22], pixel-based classification [23], and scene understanding [24]. Various research projects on automated object extraction have also been conducted. Yang et al. [25] proposed a new network dependent on DenseNets networks and the attention mechanism for the rational use of functions at various levels. X. Li et al. [26] designed a new deep opponent network named Building-A-Nets, which uses the opponent’s structure as a solid segmentation of the roofs of buildings. L. Li et al. [27] presented a new model of CNNs called a Multiple-Feature Reuse Network (MFRN) in order to reduce the requirements for GPU memory. Lu et al. [28] used richer convolutional features (RCFs) to detect edges of objects based on remote sensing images with high spatial resolution. Bittner et al. [29] developed a fully convolutional network (FCN), which effectively combines high-resolution images with normalised DSM and automatically generates prognoses for the objects. Xu et al. [30] extracted objects from high-resolution remote sensing images with use of the Res-U-Net deep learning architecture and directed filters. Boonpook et al. [31] applied the SegNet deep learning architecture to build extraction from very high-resolution imagery from unmanned aerial vehicles (UAVs). H. Liu et al. [32] proposed a fully convolutional network (DE-Net) that was created to store information with the use of network calculations, especially in down-sampling, encoding, and up-sampling procedures.

### 2.2. Research Purpose

This study attempts to verify the following research hypothesis: the detection of atypical aviation obstacles with the use of a deep neural network whose structure is based on the YOLO architecture, introducing a new algorithm for the classification of point clouds, which is adapted to the geometrical features of atypical aviation obstacles and the criterion for the filtration of a point cloud obtained from a low altitude enabling the detection of aviation obstacles with an accuracy that meets the requirements of ICAO regulations [33,34,35,36].

The aim of the research was to develop a methodology for the automated detection and classification of atypical aviation obstacles based on the data obtained from unmanned aerial vehicles.

The paper is structured as follows: in Section 3, the research method is explained. Section 4 presents test data and the experiment results. In Section 5, the results are discussed. Finally, Section 6 provides a brief summary of this work.

## 3. Methods

**Figure 1:**
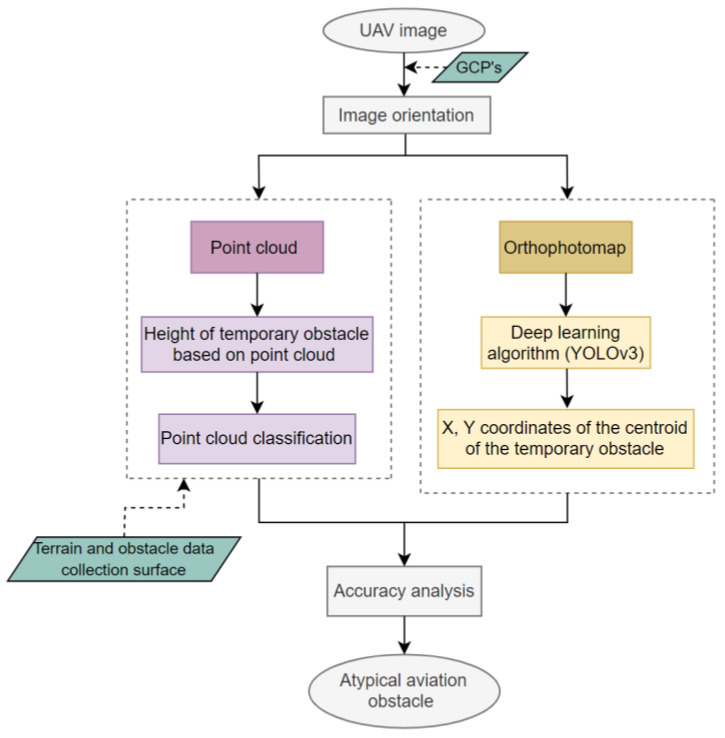
The scheme of detection of the atypical aviation obstacles.

This section describes the methodology of detecting atypical aviation obstacles based on data obtained from UAVs. The whole process is presented in the block diagram below (Figure 1). The diagram illustrates the essential stages of the developed methodology of detecting atypical aviation obstacles. The first stage consisted in obtaining the photos from the unmanned aerial vehicle. The next step was photogrammetric processing in Pix4D. As a result, a point cloud and an orthophotomap were obtained. They were then the basis for further analyses. The orthophotomap was used to detect obstacles in the image with the use of the YOLO algorithm, which is based on a convolutional neural network (CNN) [14]. The objects detected in the orthophotomap were the basis for the determination of the *x*, *y* coordinates of the centroid *C_i_* for each atypical obstacle. The previously generated point cloud was then used to determine the height of the obstacles. Based on the *x*, *y* coordinates of the objects that were determined with the use of the YOLO algorithm, these coordinates were defined in the point cloud. As a result, the search area in the point cloud was narrowed. Later, areas for collecting data about obstacles were generated and used to determine which of the detected objects penetrate through the surface of the areas and thus become aviation obstacles [37]. A new algorithm was used to determine the height of atypical aviation obstacles based on the point cloud. It assumed an iterative search of the point cloud in reference to the determined centroid in order to determine the height *H_max_*. The accuracy of the developed method was analysed based on the conducted experiments and data about aviation obstacles contained in the Aeronautical Information Publication (AIP), in the supplements that contain information about temporary aviation obstacles in the vicinity of airports. Both the horizontal coordinates *x*, *y* and the vertical *H* coordinate were analysed.

### 3.1. Detection of Atypical Aviation Obstacles Using YOLOv3

**Figure 2:**
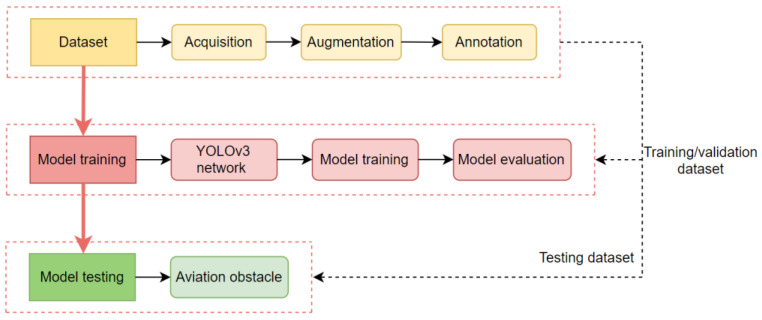
Overall architecture of the proposed methodology.

The detection of atypical aviation obstacles based on image analysis was performed with use of the YOLOv3 algorithm. The general course of detecting objects in an image is presented in Figure 2. Data obtained from UAVs were subjected to photogrammetric processing. As a result, an orthophotomap was obtained. This orthophotomap then became the source of data for creating a set of data about atypical aviation obstacles. The objects in the image were enclosed in envelopes. After the YOLO network was optimised and trained on the developed datasets, the effectiveness of detection was checked. Finally, the best model of selecting atypical aviation obstacles in an orthophotomap was selected.

The YOLOv3 algorithm employs convolutional neural networks for the detection of objects. Neural networks separate the features from images by layers of the convolution and use the fully connected layers to predict the probability of output and information about the position of the limiting rectangle. The main advantage of the algorithm as a single-stage approach is the fact that the whole image is assessed by a single neural network. It generates all prognoses based on the actual image, instead of the proposed regions, as it is done in two-stage methods. The input image is represented as a tensor of the dimensions n × m × 3, where n and m refer to the width and height in pixels, and 3 refers to three colour channels. The YOLOv3 algorithm was created based on the YOLOv2 algorithm, which had a relatively low accuracy of detecting small objects. Due to that, certain improvements were introduced, resulting in the new version of the algorithm: YOLOv3. Firstly, the algorithm performs classification with numerous labels [38], where independent logistic classifiers are used instead of the softmax classifier to predict classes with multiple labels. In the learning phase, YOLOv3 uses binary cross-entropy loss instead of the general mean square error to predict classes. A different bounding box prediction was presented in the work of Al-Saffar et al. [39], where the objectness score is set to 1 if the bounding box prior overlaps a ground truth object more than others. However, if the bounding box prior overlaps a ground truth object by more than a chosen threshold, the prediction is ignored. Therefore, YOLOv3 has only one bounding box anchor for each ground truth object. The work of Y. Li et al. [40] presented predictions across scale, where YOLOv3 can predict boxes on three different scales and then extracts features from those scales using feature pyramid networks.

The YOLO algorithm is an end-to-end network, so the whole process uses the method of calculating loss which is referred to as the sum-squared error [41]. It is a simple sum of the differences, including coordinate errors, IoU (Intersection-over-Union) errors, and classification errors. YOLOv3 uses the Darnket-53 convolutional skeleton that consists of 53 convolutional layers, where it uniformly samples the input image to the dimensions 416 × 416 and assumes that the image is divided into 3 × 3 grids.

### 3.2. Determination of the Centroid of an Atypical Aviation Obstacle

Detecting the temporary aviation obstacles with use of the YOLOv3 algorithm allowed for the determination of the *x, y* coordinates of the centroid *C_i_* of each obstacle. The determination of centroid *C* for the obstacle, whose cross-section was a non-intersecting polygon defined by *N* vertices (*x*_0_, *y*_0_), (*x*_1_, *y*_1_), …, (*x_n−_*_1_, *y_n−_*_1_) at the point (*Cx*, *Cy*), was calculated using the following formula:

(1) $$C_{x}=\frac{1}{6A}\overset{n-1}{\underset{i=0}{\sum }}\left(x_{i}+x_{i+1}\right)(x_{i}y_{i+1}-x_{i+1}y_{i})$$

(2) $$C_{y}=\frac{1}{6A}\overset{n-1}{\underset{i=0}{\sum }}\left(y_{i}+y_{i+1}\right)\left(x_{i}y_{i+1}-x_{i+1}y_{i}\right)$$

where *A* is the signature area of the polygon

(3) $$A=\frac{1}{2}\overset{n-1}{\underset{i=0}{\sum }}(x_{i}-x_{i+1}y_{i})$$

The *x*, *y* coordinates of the *C_i_* centroid of the obstacles, whose cross-section is an ellipse with extremes *A_i_*, *B_i_* of known *x*, *y* coordinates, were calculated using the following formula:

(4) $$C=\left(\frac{x_{A}+x_{B}}{2},\frac{y_{A}+y_{B}}{2}\right)$$

where: *x_A_*, *y_A_* are coordinates of the extreme point of ellipse *A*, and *x_B_, y_B_* are coordinates of the extreme point of ellipse *B*.

The determination of the coordinates of the centroid of each detected obstacle was essential for the subsequent stage of analysis, i.e., determining the heights of the aviation obstacles.

### 3.3. Estimation of Height of Temporary Aviation Obstacle

**Figure 3:**
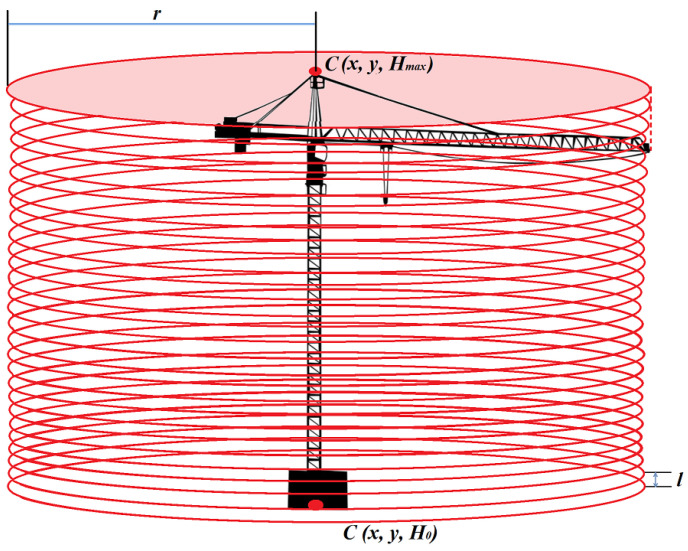
Determination of the centroid of the detected obstacle.

The determination of the *H_max_* height of an atypical obstacle consisted in an interactive search of the point cloud with the aim to detect the highest point that belonged to the analysed object. The subjects of the analysis were atypical aviation obstacles, which include, among others, construction cranes, wind turbines, energy poles, and masts. In order to define the height of a construction crane, data about the centroid of obstacle *C (x, y)* were used and the maximum radius *r* of the crane boom reach was determined (Figure 3).

The maximum value of the *r* radius was calculated using the data that define the technical parameters of this type of objects. According to the assumptions, the maximum value of the *r* radius is 90 m.

The first stage of searching the point cloud consisted in the determination of the initial height *H*_0_ in the point cloud. For the centroid *C* of the obstacle, of the known coordinates *x*, *y*, the *S_i_* plane with the *r* radius was defined:

(5) $${\left(x-a\right)}^{2}+{\left(y-b\right)}^{2}=r^{2}$$

The realisation of the algorithm (iteration *i* = 1) started with searching the *S_i_* plane for the height *H*_0_. The found point *A* of the point cloud belonged to the dataset *B* of the point cloud of the temporary obstacle. The next stage (*i* = 2) was conducted at the distance *l* = *H*_0_ + 10 cm. Each subsequent iteration was performed for the *S_i_* plane situated 10 cm above the preceding one. The height search was continued until the last point belonging to the set of point cloud *B* was found.

(6) $$H_{max}\to A\in B,B\geq 1$$

**Figure 4:**
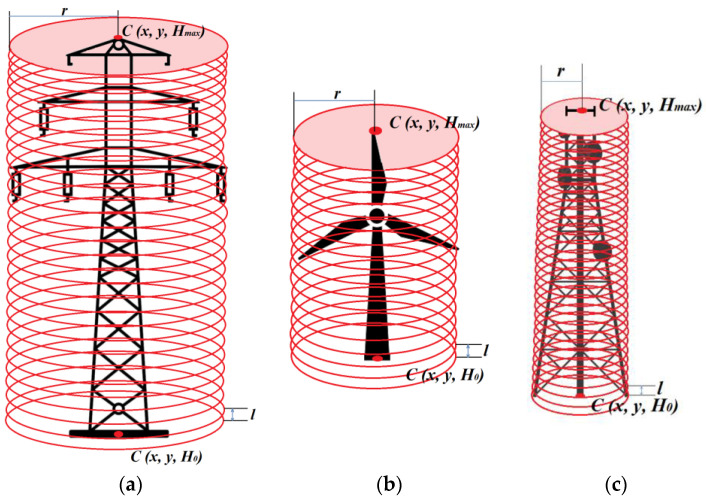
Determination of the height of the detected obstacle: (**a**) energy pole, (**b**) wind turbine, (**c**) mast.

The height of other types of obstacles (wind turbines, energy poles, and masts) was determined in a similar way as the height of the construction crane. The *x*, *y* coordinates of the *C* centroid of the obstacle and the radius *r* of the reach of the analysed obstacle were used (Figure 4). The value of the *r* radius was defined based on the maximum technical parameters of the given type of object. In the subsequent step, the iterative search of the point cloud was performed with the aim to determine the height of the obstacles *H_max_*.

### 3.4. Classification of Point Cloud

**Figure 5:**
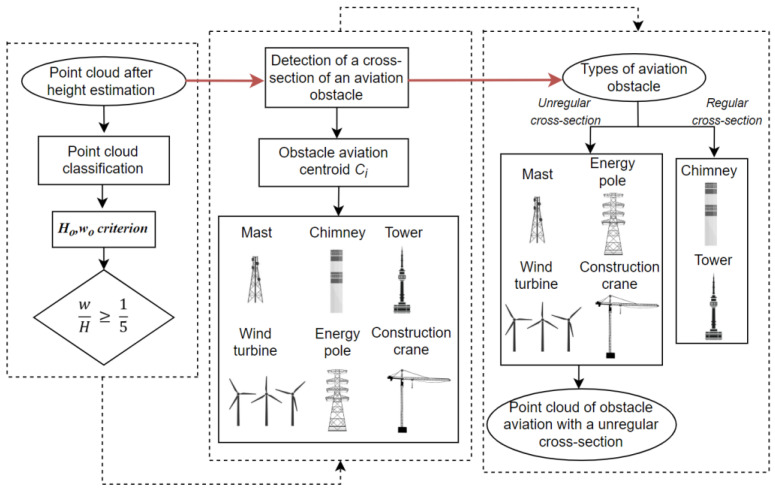
Scheme of classification of point cloud.

The aim of the classification of point cloud was to detect aviation obstacles that belong to the group of atypical obstacles. The course of the classification procedure is presented in the diagram below (Figure 5). It was assumed that the characteristic features that define atypical obstacles are their elongated shape and irregular cross-section. The first adopted classification criterion was the height of the objects which was determined based on the point cloud. The second criterion was based on the distribution of the points in the point cloud in relation to the centroid *C_i_* of the analysed object.

It was assumed that for objects belonging to the group of elongated obstacles, the ratio of the width *w* of the object to its height *H* [42] should meet the following condition:

(7) $$\frac{w}{H}\geq \frac{1}{5}$$

where: *w* is the width of the obstacle and *H* is the height of the obstacle.

The ranges of obstacle classification considering their width and height were defined with a confidence interval of 68%.

**Figure 6:**
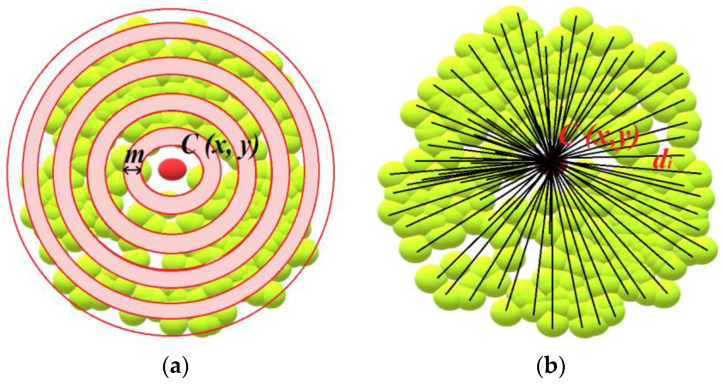
Analysis of the distribution of the points in the point cloud in relation to centroid *C_i_*: (**a**) Searching the point cloud in belts *p_i_* of a fixed width *m*; (**b**) The determination of the position of the points of the point cloud in *p_i_* belts based on the determined distance *di* of the points in the cloud from the centroid *C_i_*.

Then, the point clouds assigned to the relevant groups of obstacles were classified taking into account their cross-section, where the characteristics of the distribution of points in the point cloud in relation to the centroid *C_i_* were analysed. To this end, an iterative search of the point cloud was conducted in belts *p_i_* of a fixed width *m* (Figure 6a). The position of the points of the point cloud in *p_i_* belts was calculated based on the determined distance *di* of the points in the cloud from the centroid *C_i_* (Figure 6b)*,* using the following formula:

(8) $$d_{i}=\sqrt{{\left(x_{C}-x_{i}\right)}^{2}+{\left(y_{C}-y_{i}\right)}^{2}}$$

where: *x_c_* is the coordinate *x* of the centroid [m], *y_s_* is the coordinate *y* of the centroid [m], *x_i_* is the coordinate *x* of the point in the point cloud [m], and *y_i_* is the coordinate *y* of the point in the point cloud [m].

The maximum number of iterations and searches of the point cloud was calculated based on the ratio between the maximum distance *d_max_* of the point in the point cloud from centroid *C_i_* to the width *m* of the *p_i_* belt, using the following formula:

(9) $$i_{max}=\frac{d_{max}}{m}$$

## 4. Materials and Experimental Results

### 4.1. Study Area

**Figure 7:**
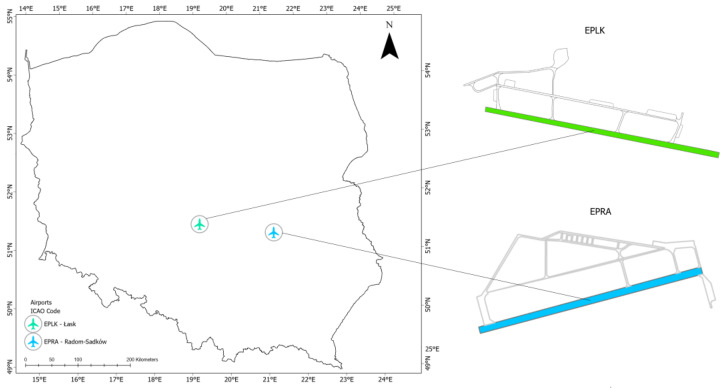
Location of the research areas.

The research was conducted at two test sites, located in the direct vicinity of the Łask military airport (ICAO code: EPLK) and the Radom–Sadków airport (ICAO code: EPRA) (Figure 7). The Łask airport (ARP: 51°33′04″ N; 019°10′57″ E) is situated in central Poland, while the Radom–Sadków airport (ARP: 51°23′20″ N; 021°12′42″ E) is located east of the Łask airport.

### 4.2. Description of Data Sets

#### 4.2.1. EPLK

The source data for the generation of the dense point cloud was obtained using the Trimble UX-5 airframe, equipped with a Sony a7R camera. The aerial platform was equipped with a single-frequency GPS receiver, recording data at the frequency of 10 Hz.

Flights were conducted in two test areas in April 2019. The photographic conditions were good, i.e., the sky was covered with a small amount of cumulus clouds, and the average wind velocity was approximately 2 m/s. Camera settings were defined in the manual mode, while the focus of the lens was set to infinity. The first and the second test sites were the areas surrounding the Łask military airport. The measurement campaign consisted of 15 test blocks, where each block contained about 600 images. The data were obtained at the altitude of approx. 250 m above the ground level. Flights were performed in the East–West direction, based on the assumption that the longitudinal and transverse coverage was approx. 75%. The signalled photopoints were designed and measured in the test area. All the points were measured with use of the RTK technique in the GNSS system. The terrain coordinates of the photopoints were determined with the mean error of m_x,y,z_ = ±0.03 m. The first area contained six photopoints and eight independent control points, while for the second the numbers were, respectively, six and seven. The ground sampling distance (GSD) was 0.04 m.

#### 4.2.2. EPRA

The source data for aerotriangulation was obtained using the VTOL WingtraOne system, equipped with a Sony RX1R II camera. The aerial platform was equipped with a single-frequency GPS receiver, recording data at the frequency of 10 Hz. As part of the research analysis, the GNSS data recorded by an AsteRx-m2 UAS receiver placed on the Tailsitter unmanned platform was used. The flight was carried out in the two research areas in June 2021. The imaging conditions were good. The first and the second research area covered the area around the Radom–Sadków airport. Fourteen test blocks were realised during the flight, and each of them contained almost 600 images. The data were obtained at the altitude of 250 m above the ground level. The flight was conducted in the East–West direction, assuming that the transverse and longitudinal coverage was 75%. The signalled photopoints were designed and measured with use of the RTK technique in the GNSS system in the test area. The terrain coordinates of the control points were determined with the mean error m_x,y,z_ = ±0.03 m. The first area contained six photopoints and seven independent control points, while for the second the numbers were, respectively, four and seven. The ground sampling distance (GSD) was 0.04 m.

### 4.3. Atypical Aviation Obstacles

Objects that may pose a threat to aerial vehicles due to their dimensions are referred to as aviation obstacles. Apart from permanent objects such as buildings or terrain elevations, aviation obstacles may also be fixed objects of a temporary nature, or mobile objects (e.g., construction cranes), which are called temporary aviation obstacles. Obstacles whose height exceeds the limiting planes, are considered to be objects that may pose a threat in the aviation space. Temporary objects, such as construction cranes, are slender structures, and their atypical shape makes it more difficult to determine their height. Permanent obstacles may also have the form of elongated or slender objects, which include, among others: masts, wind turbines, chimneys, and energy poles (Table 1).

### 4.4. Surfaces of Obtaining Data about Obstacles

Ensuring safety in the aviation space is a very complex process. Some of its numerous elements include creating aviation maps, designing procedures, etc. The basis for creating aviation materials is obtaining and collecting data about aviation obstacles.

As a result of the need to gather and store such data, the following coverage areas are distinguished: 1, 2 (2a, 2b, 2c, 2d). Area 2a is a rectangular area around the runway that includes the runway itself and the abandoned take-off security area, if it exists. Area 2b is the area that stretches from the end of area 2a in the direction of take-off, 10 km long and opening at an angle of 15% to each side. The inclination of this area is 1.2%. Area 2c stretches outside areas 2a and 2b to a maximum of 10 km from the border of area 2a. Its surface has an inclination of 1.2%. Finally, area 2d is an area located outside areas 2a, 2b, and 2c, which reaches up to 45 km from the reference point of the airport or to the existing TMA border; whichever is closer [37].

**Figure 8:**
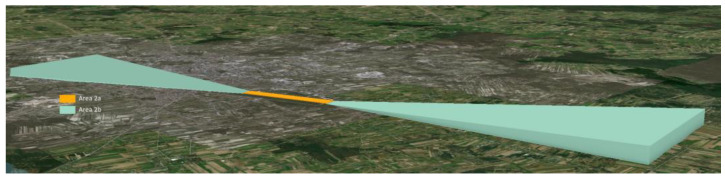
Obstacle data collection surfaces—Area 2a and area 2b (side profile view).

Collecting data on obstacles in areas 2a and 2b (Figure 8) is directly related to ensuring safety in the airspace during the critical phases of the aircraft’s flight, i.e., take-off and landing. Therefore, it is necessary to update the data on obstacles in these areas on an ongoing basis.

Areas 2b and 2c are inclined planes that rise starting from area 2a. These areas determine the threshold altitudes for collecting data about aviation obstacles. The most important data about the obstacles are their horizontal location, height, and type of obstacle.

### 4.5. Experimental Results

**Figure 9:**
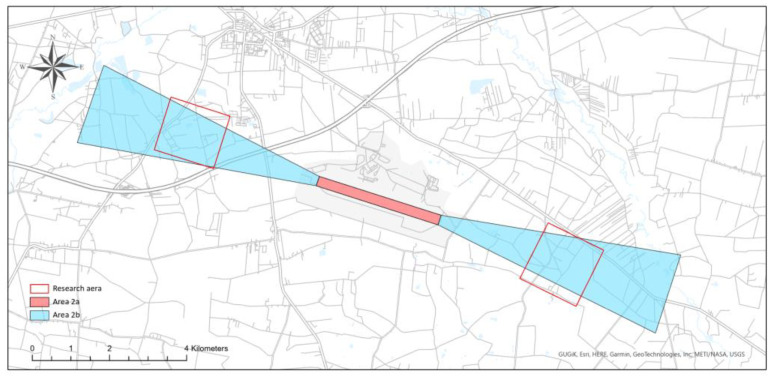
Test areas—Łask airport.

**Figure 10:**
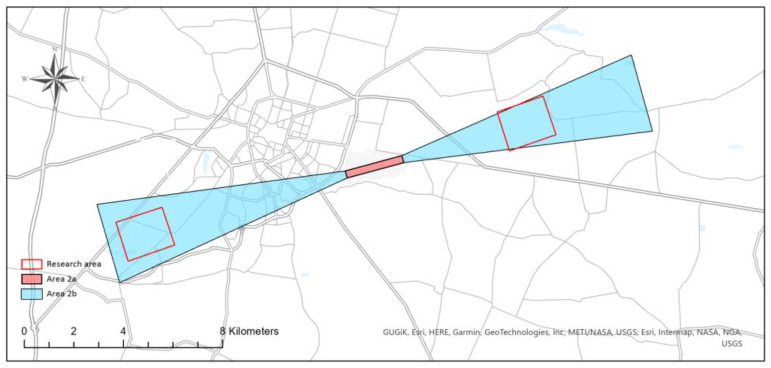
Test areas—Radom–Sadków airport.

The experiments were conducted in two test areas located in the vicinity of the Łask airport (EPLK) and two test areas near the Radom–Sadków airport (EPRA). Tests were conducted on data obtained from UAVs during four photogrammetric flights. Two of them took place in the direct vicinity of the Łask airport (Figure 9) and the other two near the Radom–Sadków airport (Figure 10). The test areas were selected based on two criteria: the first one assumed that the study area should match the 2b area, i.e., the area of collecting data about terrain and obstacles. The second criterion was the existence of tall objects that might interfere with safety in the aviation space. The test areas were selected so as to gather the largest possible amount of data.

At the first stage, images were obtained from the UAVs and then processed in specialist Pix4D software. During the photogrammetric data processing, a dense point cloud was generated, which was later used to determine the height of atypical aviation obstacles. Apart from that, an orthophotomap was created and used to detect atypical aviation obstacles with use of the YOLOv3 algorithm and to define their location. The detection of atypical aviation obstacles started with the analysis of the orthophotomap. To this end, artificial neural networks were used to separate the features from the images. The *C_i_* centroids were defined for each detected object. The data about the location of atypical aviation obstacles obtained in this way made it possible to find these objects in the point cloud and were the starting point for further analyses of the point cloud.

### 4.6. Detecting Atypical Aviation Obstacles in the Orthophotomap

At this stage, the YOLOv3 algorithm based on convolutional neural networks was used to detect atypical aviation obstacles. Artificial neural networks separate the features from the images by layers of the convolution and use the fully connected layers to predict the probability of output and information about the position of the limiting rectangle.

Although several trained YOLO networks containing some known datasets exist, the neural network still requires training to improve its precision in working with such specific objects as atypical aviation obstacles.

The model was trained with the use of Google Colab. A notebook based on YOLOv3 that employs trained Darknet-53 weights was used. A set of data for atypical aviation obstacles was added to the notebook. The training parameters recommended by the authors of the solution were used for network training purposes. Additionally, the training consisted of 400 epochs, which took approx. 120 min.

The network was trained for four classes of objects: construction crane, energy pole, wind turbine, and mast. This resulted in the size of the first scale output tensor of 13 × 13 × 16.

Non-standard image databases containing objects from the following classes: construction crane, energy pole, wind turbine, and mast were prepared based on the orthophotomaps being a part of the digital database for Poland created by the National Geodetic and Cartographic Resource (PZGiK). Orthophotomaps in the standard 0.10 m × 0.10 m were used for tests. The database for the wind turbine class of elevators was enriched with a publicly accessible database [43].

Most of the images used contained more than one object for the three classes: construction crane, energy pole, and wind turbine. On the other hand, in the mast class, a majority of the images contained single objects representative of the class. Objects from all classes were fully placed inside boxes. Images, where only a part of the object was visible, were excluded from the training set or the object was not marked as belonging to a specific class.

The research experiment was conducted on 800 images that contained a total of 1023 objects. The images were randomly divided into three sets of data: training data (70% images) used to estimate the weights of the artificial neural network, validation data (20% images) used to test the trained network, and test data (10% images) used to test the functioning of the network after training. The set of training data consisted of 560 images that contained a total of 716 objects, the validation data set consisted of 160 images containing a total of 205 objects, while the test data set consisted of 80 images containing a total of 102 objects. The full distribution of the data set is presented in the table below (Table 2).

**Figure 11:**
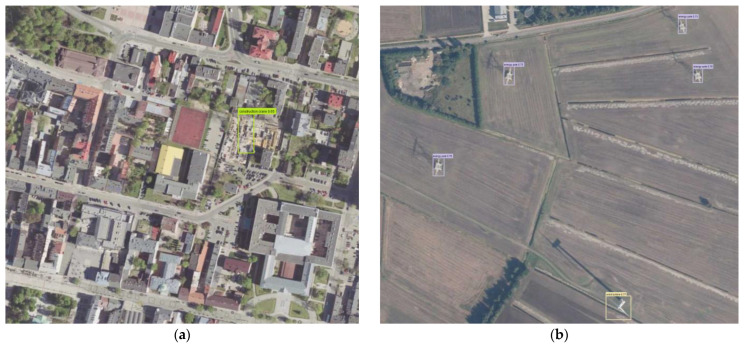

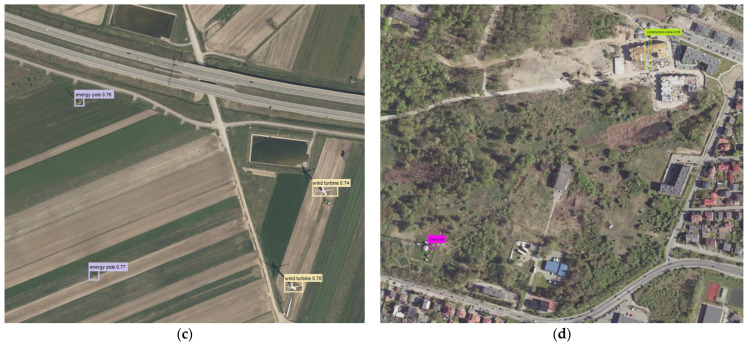
Sample detection (fragment of orthophotomap): (**a**) construction cranes; (**b**) wind turbines and energy poles; (**c**) wind turbines and energy poles; (**d**) construction crane and mast.

The results of the detection of atypical aviation obstacles on the data from the test dataset are presented in Figure 11a–d. A sample limiting box for the construction crane class, in green, is presented in Figure 11a,d. A sample limiting box for the energy pole class, in pink, is presented in Figure 11b,c. A sample limiting box for the wind turbine class, in beige, is presented in Figure 11b,c. A sample limiting box for the mast class, in magenta, is presented in Figure 11d.

The detection of atypical aviation obstacles in the orthophotomap allowed for the determination of the centroid *C (x, y)* for each detected object. Data about the location of aviation obstacles obtained in this way enabled research conduction on the point cloud.

### 4.7. Accuracy Evaluation of YOLOv3 Algorithm

The indicators used to assess the accuracy of the detected aviation obstacles are average precision (*AP*) and mean average precision (*mAP*). *AP* defines the proportion of the correct detections to the sum of the correct detections (i.e., the correct determination of the location and classification) and false detections of objects. A high value of the *AP* coefficient means that there are few false predictions. The *mAP* coefficient is used to measure the average accuracy of detection of multiple types of objects. The higher the *mAP*, the more comprehensive the model is in all categories. Average precision and mean average precision are calculated using the formula below [44]:

(10) $$AP=\overset{N}{\underset{k=1}{\sum }}Precision(k)\Delta Recall\left(k\right)$$

(11) $$mAP=\frac{1}{N}\overset{N}{\underset{i=1}{\sum }}AP_{i}$$

where: *AP_i_* is the average accuracy of class *i,* and *N* is the number of classes.

During the measurement of *mAP*, the efficiency of both the classification and the positioning with the use of limiting frames in the image are assessed. The *mAP* formula is based on Confusion Matrix, Intersection over Union (IoU), and Recall and Precision.

Objects are detected based on the Intersection over Union (IoU) concept [45,46]. IoU measures the overlapping of two borders and is used to estimate the extent to which the predicted border overlaps with the actual border of the object. The IoU value is a measure of the accuracy of the determination of the position and size of the object. The measure is calculated based on the actual frame of the object and the frame returned by the artificial neural network. It is defined as the ratio of the product (intersection of the boxes) to their sum.

Based on the data in Table 3, it was found that the value of the IoU index for the construction crane class was 69.4%, for the energy pole it was 78.2%, for the wind turbine class it was 74.2%, and, finally, for the mast class it was 64.9%. These results demonstrate that the accuracy of the positioning and location was better for objects belonging to the energy pole and wind turbine classes. However, the IoU index for the construction crane and mast classes was slightly lower. The value of the average precision (*AP*) for the construction crane category was 74.8%, for the energy pole class it was 67.6%, for the wind turbine class it was 65.2%, and for the mast class 75.3%. A high value of the *AP* coefficient means that few false predictions were obtained. The *mAP* value of the applied YOLOv3 algorithm reached the value of 70.7%. Such relatively high value of *mAP* confirms that the model is comprehensive and that this algorithm may be used to detect atypical aviation obstacles.

**Figure 12:**
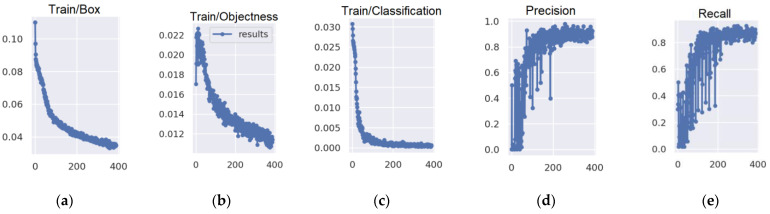

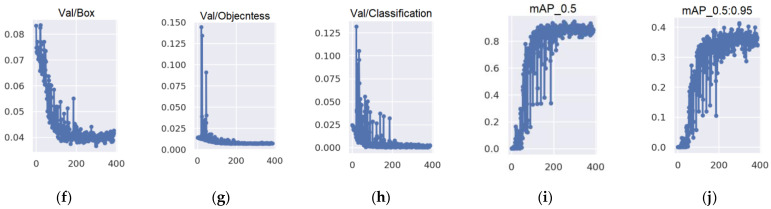
Accuracy evaluation curves: (**a**) Box loss function in the training process; (**b**) Objectness loss function in the training process; (**c**) Classification loss function in the training process; (**d**) Precision; (**e**) Recall; (**f**) Box loss function in the validation process; (**g**) Objectness loss function in the validation process; (**h**) Classification loss function in the validation process; (**i**) *mAP* when IoU is set to 0.5; (**j**) *mAP* when IoU is set from 0.5 to 0.95.

One may distinguish three different types of loss: box loss, objectness loss, and classification loss. The box loss shows how well the algorithm is able to locate the centre of the object and how well the predicted bounding box covers the object, while objectness is, in general, a measure of the likelihood of the presence of the object in the proposed area of interest. Finally, the classification loss provides an idea of how well the algorithm can predict whether the given object belongs to a class. The curves of loss of the applied YOLOv3 algorithm for 400 epochs are presented in Figure 12a–h. For the training data, the box loss and objectness loss diagrams show high fluctuations for the first half of the epochs. After approx. 200 epochs, the curve stabilises. The classification loss curve flattens after about 50 epochs. For validation data, the box loss curve begins to stabilise after 200 epochs, while the objectness loss curve stabilises after about 50 epochs. The course of the classification loss curve for validation data is very dynamic in the initial epochs. Then, it stabilises after approx. 200 epochs. Based on these curves, it was found that the loss of the model decreases gradually with the increase in the number of epochs. Recall and precision curves (Figure 12d,e) stabilise after 200 epochs and demonstrate that the model is reliable. The *mAP* diagram for IoU = 0.5 (Figure 12i) shows that the model stabilises after 200 epochs, giving a high *mAP* index. The dynamics of the course of the *mAP* curve for IoU = 0.5–0.95 (Figure 12j) is similar. However, in this case the value of the *mAP* index is lower.

Based on the obtained results, it was found that the *mAP* index achieved higher values for lower values of the IoU (Figure 12i), which means that the boxes are not perfectly matched to the dimensions of the objects. The system obtained poorer results for small objects that belonged to the mast class and for objects of an elongated shape in the image which belonged to the construction crane category. This was an expected result, as one of the characteristic properties of the YOLO network is fast operating speed, but at the expense of a slightly worse detection of small objects. Additionally, it was noted that the efficiency of the network was lower for objects from the classes mentioned above, which were mostly situated in densely developed areas. Slightly lower values of the *mAP* index were achieved for higher values of IoU. In this group, obstacles belonging to the energy pole and wind turbine classes were detected. It was noted that a high value of the *mAP* index confirms that the model is comprehensive and that this algorithm may be used to detect atypical aviation obstacles.

### 4.8. Detection of Atypical Aviation Obstacles Based on Point Cloud

Atypical aviation obstacles were classified based on the point cloud. It was assumed that such atypical obstacles are characterised by a slender, elongated shape, and an irregular cross-section. These characteristics were the basis for the determination of the classification criteria of atypical aviation obstacles. The first criterion was the height of the analysed objects, which was determined based on the point cloud. The second criterion was the geometric properties of the point cloud in transverse cross-section in reference to the defined centroid *C_i_* of the object.

#### 4.8.1. Generating a Dense Point Cloud

The photos obtained during UAV flights were subjected to photogrammetric processing in the Pix4D software. The matching of multiple images made it possible for the research team to obtain a “dense” cloud of points. The input data for generating the point cloud were the images and their external orientation. Based on the obtained point cloud, the RMS error of the position of the 3D point was determined. For the first test area it was 0.4 m, for the second test area it was 0.3 m, for the third test area it was 0.3 m, and for the fourth test area it was 0.2 m.

#### 4.8.2. Classification of Point Cloud

**Figure 13:**
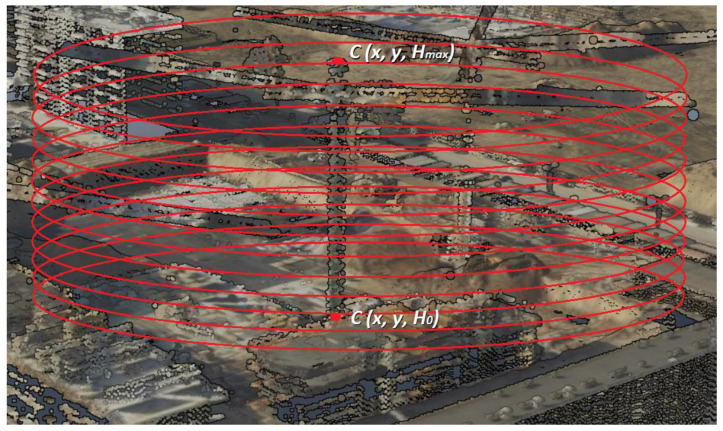
Classification of construction crane point cloud.

The detection of atypical aviation obstacles conducted on the orthophotomap enabled to determine their location (*C (x, y)*). The data of the horizontal coordinates *x*, *y* of aviation obstacles were used to locate these objects in the point cloud. The classification of the cloud was performed with use of the iterative method for each previously located aviation obstacle. The determination of the *H_max_* height of an atypical obstacle consisted in an iterative search of the point cloud with the aim to detect the highest point that belonged to the analysed object. The initial stage of point cloud search was performed for *C* (*x*, *y*, *H*_0_), where *H*_0_ is the minimum height of the object determined based on the point cloud (Figure 13). Iterations were performed until the last point belonging to the object was found in the point cloud.

The classification of the point cloud was followed by its filtration. The aim of this stage was to analyse the previously detected objects based on the heights obtained from the point cloud. To achieve it, areas 2a and 2b were created to collect data about obstacles for the Radom–Sadków and Łask airports. Area 2a was a flat plane, while area 2b was a plane inclined by 1.2% in relation to area 2a.

**Figure 14:**
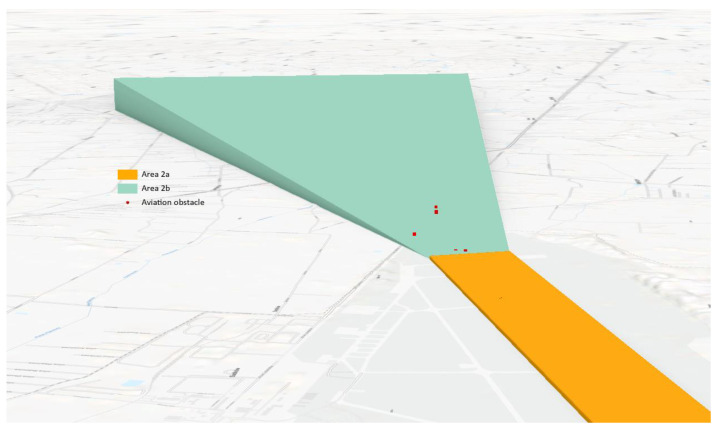
Area 2a and area 2b with atypical aviation obstacles—Airport Radom–Sadków.

The generated planes constituted the border above which data on aviation obstacles are collected. Plane 2b rose to the specified height *H.* The data concerning height allowed for the filtration of the point cloud. The points from the point cloud that penetrated through the plane (Figure 14) automatically became obstacles and were included in the set of obstacles’ data.

### 4.9. Analysis of the Matching Accuracy of the Point Cloud

The matching accuracy of the point cloud was analysed based on the reference data contained in the database on obstacles, as well as in the supplements attached to the Aeronautical Information Publication. The supplement contains, among others, information about temporary aviation obstacles. The obstacles’ database is developed taking into account all reported aviation obstacles. It contains information concerning: the geographical latitude and longitude of the obstacle, its absolute height, elevation above ground level, location (town), type of obstacle, etc. The analysis of the matching accuracy of the point cloud took into consideration how well it fit in the horizontal plane (coordinates X, Y) and in the vertical plane (coordinate Z) (Table 4).

The accuracy analysis was conducted for four classes of aviation obstacles: construction crane, energy pole, wind turbine, and mast.

The analysis of the statistical values that characterise the matching accuracy of the point cloud revealed that the average difference in horizontal coordinates (X, Y) fell into the range from 0.3 m to 0.7 m, while the average difference in height (Z) ranged from 0.4 m to 0.6 m. The average error of matching between the point cloud and the set of data about obstacles ranged from ±0.4 m to ±0.7 m, whereas the standard deviation was between 0.5 m and 0.6 m. The results of the accuracy analysis demonstrated that the method of detection and classification of aviation obstacles guarantees horizontal and vertical accuracy at the level of several tens of centimetres.

## 5. Discussion

Section 4, which presents the research experiments and the results of detecting atypical obstacles, confirms the high efficiency of the developed method. That section discussed the complexity of the process of accurate detection of aviation obstacles. The complexity consisted in combining two different techniques of obtaining data: from the image, with the use of the YOLOv3 algorithm, and as a result of the point cloud analysis. The latter consisted in an iterative search of the point cloud in reference to the previously defined centroid in order to determine the height. The point cloud matching results met the accuracy requirements provided in the ICAO documentation [33,34,35,36]. The results of the detection of atypical aviation obstacles demonstrated that the performance of the neural network determined with the use of the *mAP* index was better for objects where the boxes were less accurately matched to the dimensions of the objects. This case referred to the mast category, which was represented by small objects. Previous research on the detection of small objects based on images from UAV revealed that using the YOLOv3 algorithm to detect such objects results in lower accuracy [47]. Apart from that, a lower IoU index was noted in the construction crane category. Objects belonging to this group were characterised by a thin shape in the image. Both masts and construction cranes were usually located in densely built-up areas. A slightly lower value of the *mAP* index was found in the wind turbine and energy pole categories. However, in these cases, the value of the IoU index was higher, which proved that such objects were detected with higher accuracy. Most of them were located in open areas, free from dense development.

Although the efficiency of detecting small objects with the use of deep learning methods has improved significantly in recent years, there is still a difference between the accuracy levels achieved for small and large objects [48]. Most research studies present satisfactory results in the detection of large objects. The YOLOv3 algorithm is being continuously improved in order to enhance the efficiency of detecting small objects in images. Liu et al., in their work [47], proposed improving the darknet structure by means of increasing the convolutional operations in the early layer in order to enrich the spatial information. Some studies also revealed an effective reduction of the gap in detecting small objects by means of increasing the number of datasets that use vast amounts of data to train the models [49,50].

UAV provides the images which then become the basis for detecting various types of objects with the use of the YOLOv3 algorithm. Kharchenko et al., [51] in their research on the detection of objects in the vicinity of an airport demonstrated that the YOLOv3 algorithm was characterised by a high detection capacity and positioning accuracy. Moreover, the works by Junos, Mohamad Haniff et al. [52] showed the influence of the correction of images from UAVs on the results of the detection of objects with the use of the YOLOv3 algorithm. Similar accuracy was obtained at the level of 0.3 m to 0.5 m by Mitsevich [53], who proposed a solution for remote and effective obstacle identification and assessment processes with the use of remote sensing stereo imagery. The photogrammetric methods based on the three-dimensional vector models were used, which were integrated into the stereo pair of satellite and aviation scanner images.

The results of detection of atypical aviation obstacles were also compared with the use of methods standardly used for the acquisition of data for the needs of GIS. Wierzbicki et al. [54] have proposed a modified, fully convolutional U-Shape Network (U-Net) for the segmentation of a high-resolution aerial orthoimages and dense LiDAR data in order to automatically extract building outlines. Rottensteiner et al. [55] detected buildings with the Dempster–Shafer method using LiDAR data and aerial photos, and they reached an accuracy of 85%. Sohn and Dowman [56] achieved a building detection accuracy of 80.5% using a combination of IKONOS and LiDAR satellite data. A detailed analysis of the accuracy has been presented in the work of Khoshboresh-Masouleh et al. [57], where various types of areas have been examined, reaching an IoU value of 76%. The position accuracy of the detection of buildings for the purposes of GIS ranges from 0.7 m to 1.5 m in a wide variety of research [58,59,60]. Therefore, the results of aviation obstacle detection presented in this article correspond to the results of object detection carried out by different authors using other techniques.

## 6. Conclusions

The presence of aviation obstacles in the direct proximity of the airport may have a negative influence on ensuring safety in the aviation space. The existing databases about aviation obstacles have to meet certain requirements concerning accuracy, which are provided in the legal standards that regulate international aviation law. These documents also contain the requirements and techniques for obtaining data about aviation obstacles. Although the issues related to collecting data about obstacles have been regulated, the automation of the process of collecting data about elongated obstacles still needs improvement [1]. In order to capture very thin objects, a larger image scale than one used for traditional exploratory flights is required. This, in turn, requires lower altitudes, which may be achieved by using UAVs to detect this type of obstacles. At a lower flight altitude, the obtained spatial accuracy (x, y, z) will be the highest. The automation of the process of obtaining data about obstacles, in particular elongated ones that may be described as atypical, still remains a challenge.

The paper constitutes an attempt to present the method of automated detection and classification of atypical aviation obstacles based on data obtained from UAVs. The research was conducted with the use of the YOLOv3 algorithm to detect objects in the image. The methodology of the classification of the point cloud that had been presented in the previous study by Lalak et al. [61] has also been modified. The main aim of the methodology is to detect atypical aviation obstacles in the direct vicinity of an airport by combining the image and the point cloud data.

Based on the conducted analysis of the matching accuracy of the point cloud, it was found that the average differences in horizontal coordinates *x*, *y* were, respectively, ±0.3 m and ±0.4 m, while the average difference in height was ±0.5 m. The accuracy levels obtained on the horizontal and vertical planes met the requirements contained in the ICAO regulations [33,34,35,36]. As a result of training the model with the YOLOv3 algorithm, the value of average precision (*AP*) for the construction crane class was 74.8%, for the energy pole class 67.6%, for the wind turbine class 65.2%, and, finally, for the mast class 75.3%. A high value of the *AP* coefficient means that few false predictions were obtained. The *mAP* value of the applied YOLOv3 algorithm reached the value of 70.7%. Such a high value of *mAP* confirms that the model is comprehensive and that this algorithm may be used to detect atypical aviation obstacles.

The developed methodology may significantly improve the process of analysing the operational limitations of aerial vehicles, designing procedures or creating aviation maps, as well as enhance the security in the aviation space by limiting the risk of collision of the aerial vessel with an obstacle to a minimum.

## Figures and Tables

**Table 1:** Atypical aviation obstacles.

Type of Aviation Obstacle	
Mast	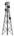
Wind turbine	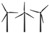
Chimney	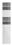
Tower	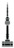
Energy pole	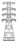
Construction crane	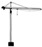

**Table 2:** Contents of the data set.

Data Set	Category	Number of Images	Number of Objects
Training set	Construction crane	143	184
	Energy pole	141	196
	Wind turbine	140	188
	Mast	136	148
Validation set	Construction crane	44	52
	Energy pole	39	63
	Wind turbine	41	49
	Mast	36	41
Test set	Construction crane	22	27
	Energy pole	19	30
	Wind turbine	21	26
	Mast	18	19

**Table 3:** Accuracy of test results in various categories.

Category	IoU (%)	*AP* (%)	*mAP* (%)
Construction crane	69.4	74.8	70.7
Energy pole	78.2	67.6	
Wind turbine	74.6	65.2	
Mast	64.9	75.3	

**Table 4:** Statistical values that characterise the matching accuracy of the point cloud.

Obstacle	Average Difference in Coordinate X (m)	Average Difference in Coordinate Y (m)	Average Difference in Height H (m)	Mean Error (m)	Standard Deviation (m)
Construction crane	0.6	0.7	0.4	0.7	0.5
Energy pole	0.4	0.3	0.5	0.5	0.4
Wind turbine	0.3	0.4	0.5	0.5	0.6
Mast	0.6	0.5	0.6	0.6	0.5

## References

[1] Eurocontrol (2021). Terrain and Obstacle Data Manual.

[2] Nex F., Remondino F. (2014). UAV for 3D mapping applications: A review. Appl. Geomat..

[3] Everaerts J. (2008). The use of unmanned aerial vehicles (UAVs) for remote sensing and mapping. Int. Arch. Photogramm. Remote Sens. Spat. Inf. Sci..

[4] Carvajal-Ramírez F., Agüera-Vega F., Martínez-Carricondo P.J. (2016). Effects of image orientation and ground control points distribution on unmanned aerial vehicle photogrammetry projects on a road cut slope. J. Appl. Remote Sens..

[5] Zein T. Fit-For-Purpose Land Administration: An implementation model for cadastre and land administration systems. Proceedings of the Land and Poverty Conference 2016: Scaling up Responsible Land Governance.

[6] Stöcker C., Bennett R., Nex F., Gerke M., Zevenbergen J. (2017). Review of the Current State of UAV Regulations. Remote Sens..

[7] Zeybek Z., Şanlıoğlu İ. (2019). Point cloud filtering on UAV based point cloud. Measurement.

[8] Gevaert C.M., Persello C., Sliuzas R., Vosselman G. (2017). Informal settlement classification using point-cloud and image-based features from UAV data. ISPRS J. Photogramm. Remote Sens..

[9] Girshick R., Donahue J., Darrell T., Malik J. Rich feature hierarchies for accurate object detection and semantic segmentation. Proceedings of the IEEE Conference on Computer Vision and Pattern Recognition (CVPR).

[10] Girshick R. Fast R-CNN. Proceedings of the IEEE International Conference on Computer Vision (ICCV).

[11] Ren S., He K., Girshick R., Sun J. Faster R-CNN: Towards real-time object detection with region proposal networks. Proceedings of the Advances in Neural Information Processing Systems 28: Annual Conference on Neural Information Processing Systems 2015.

[12] Liu W., Anguelov D., Erhan D., Szegedy C., Reed S., Fu C.Y., Berg A.C. SSD: Single shot multibox detector. Proceedings of the European Conference on Computer Vision (ECCV).

[13] Fu C.Y., Liu W., Ranga A., Tyagi A., Berg A.C. (2017). DSSD: Deconvolutional single shot detector. arXiv.

[14] Redmon J., Divvala S., Girshick R. You only look once: Unified, real-time object detection. Proceedings of the IEEE Conference on Computer Vision and Pattern Recognition (CVPR).

[15] Erhan D., Szegedy C., Toshev A., Anguelov D. Scalable object detection using deep neural networks. Proceedings of the IEEE Conference on Computer Vision and Pattern Recognition (CVPR).

[16] Simonyan K., Zisserman A. (2014). Very deep convolutional networks for large-scale image recognition. arXiv.

[17] Esetlili M., Bektas Balcik F., Balik Sanli F., Kalkan K., Ustuner M., Goksel Ç., Gazioğlu C., Kurucu Y. (2018). Comparison of Object and Pixel-Based Classifications for Mapping Crops Using Rapideye Imagery: A Case Study of Menemen Plain. Int. J. Environ. Geoinformatics.

[18] Çelik O., Gazioğlu C. (2020). Coastline Difference Measurement (CDM) Method. Int. J. Environ. Geoinformatics.

[19] Long J., Shelhamer E., Darrell T. Fully convolutional networks for semantic segmentation. Proceedings of the IEEE Conference on Computer Vision and Pattern Recognition (CVPR).

[20] Lin J., Jing W., Song H., Chen G. (2019). ESFNet: Efficient Network for Building Extraction From High-Resolution Aerial Images. IEEE Access.

[21] Huang W., Xiao L., Wei Z., Liu H., Tang S. (2015). A new pan sharpening method with deep neural networks. IEEE Geosci. Remote Sens. Lett..

[22] Chen X., Xiang S., Liu C.L., Pan C.H. (2014). Vehicle detection in satellite images by hybrid deep convolutional neural networks. IEEE Geosci. Remote Sens. Lett..

[23] Hu W., Huang Y., Wei L., Zhang F., Li H. (2015). Deep convolutional neural networks for hyperspectral image classification. J. Sens..

[24] Zhang F., Du B., Zhang L. (2016). Scene classification via a gradient boosting random convolutional network framework. IEEE Trans. Geosci. Remote Sens..

[25] Yang H., Wu P., Yao X., Wu Y., Wang B., Xu Y. (2018). Building extraction in very high resolution imagery by dense-attention networks. Remote Sens..

[26] Li X., Yao X., Fang Y. (2018). Building-A-Nets: Robust Building Extraction from High-Resolution Remote Sensing Images With Adversarial Networks. IEEE J. Sel. Top. Appl. Earth Obs. Remote Sens..

[27] Li L., Liang J., Weng M., Zhu H. (2018). A multiple-feature reuse network to extract buildings from remote sensing imagery. Remote Sens..

[28] Lu T., Ming D., Lin X., Hong Z., Bai X., Fang J. (2018). Detecting building edges from high spatial resolution remote sensing imagery using richer convolution features network. Remote Sens..

[29] Bittner K., Adam F., Cui S., Körner M., Reinartz P. (2018). Building footprint extraction from VHR remote sensing images combined with normalized DSMs using fused fully convolutional networks. IEEE J. Sel. Top. Appl. Earth Obs. Remote Sens..

[30] Xu Y., Wu L., Xie Z., Chen Z. (2018). Building extraction in very high resolution remote sensing imagery using deep learning and guided filters. Remote Sens..

[31] Boonpook W., Tan Y., Ye Y., Torteeka P., Torsri K., Dong S. (2018). A Deep Learning Approach on Building Detection from Unmanned Aerial Vehicle-Based Images in Riverbank Monitoring. Sensors.

[32] Liu H., Luo J., Huang B., Hu X., Sun Y., Yang Y., Zhou N. (2019). DE-Net: Deep Encoding Network for Building Extraction from High-Resolution Remote Sensing Imagery. Remote Sens..

[33] ICAO (2016). Annex 15 to the Convention on International Civil Aviation—Aeronautical Information Services.

[34] ICAO (2009). Annex 4 to the Convention on International Civil Aviation.

[35] ICAO (2002). DOC-9674, World Geodetic System-1984 (WGS84) Manual.

[36] ICAO (2018). DOC-1006, Aeronautical Information Management.

[37] ICAO (2018). Annex 14 to the Convention on International Civil Aviation.

[38] He K., Zhang X., Ren S., Sun J. Deep residual learning for image recognition. Proceedings of the IEEE Conference on Computer Vision and Pattern Recognition.

[39] Al-Saffar A.A.M., Tao H., Talab M.A. Review of deep convolution neural network in image classification. Proceedings of the 2017 International Conference on Radar, Antenna, Microwave, Electronics, and Telecommunications (ICRAMET).

[40] Li Y., Zhang H., Xue X., Jiang Y., Shen Q. (2018). Deep learning for remote sensing image classification: A survey. Wiley Interdiscip. Rev. Data Min. Knowl. Discov..

[41] Ranjbar M., Mori G., Yang W. Optimizing complex loss functions in structured prediction. Proceedings of the European Conference on Computer Vision.

[42] (1980). Praca zbiorowa, Geodezja inżynieryjna, Tom II..

[43] Kaggle Wind Turbine Detection. https://www.kaggle.com/datasets/saurabhshahane/wind-turbine-obj-detection.

[44] Afonso M., Fonteijn H., Fiorentin F., Lensink D., Mooij M., Faber N. (2020). Tomato fruit detection and counting in greenhouses using deep learning. Front. Plant Sci..

[45] He H., Garcia E.A. (2009). Learning from imbalanced data. IEEE Trans. Knowl. Data Eng..

[46] Csurka G., Larlus D., Perronnin F. What is a good evaluation measure for semantic segmentation?. Proceedings of the 24th BMVC British Machine Vision Conference.

[47] Liu M., Wang X., Zhou A., Fu X., Ma Y., Piao C. (2020). Uav-yolo: Small object detection on unmanned aerial vehicle perspective. Sensors.

[48] Nguyen N.D., Do T., Ngo T.D., Le D.D. (2020). An Evaluation of Deep Learning Methods for Small Object Detection. J. Electr. Comput. Eng..

[49] Lin T.Y., Maire M., Belongie S., Hays J., Perona P., Ramanan D., Zitnick C.L. Microsoft COCO: Common objects in context. Proceedings of the European Conference on Computer Vision.

[50] Russakovsky O., Deng J., Su H., Krause J., Satheesh S., Ma S., Fei-Fei L. (2015). Imagenet large scale visual recognition challenge. Int. J. Comput. Vis..

[51] Kharchenko V., Chyrka I. Detection of airplanes on the ground using YOLO neural network. Proceedings of the IEEE 17th International Conference on Mathematical Methods in Electromagnetic Theory (MMET).

[52] Junos M.H., Mohd Khairuddin A.S., Thannirmalai S., Dahari M. (2021). Automatic detection of oil palm fruits from UAV images using an improved YOLO model. Vis. Comput..

[53] Mitsevich L. (2020). 3D Aerodrome Obstacle Assessment Using Stereo Remote Sensing Imagery. Int. Arch. Photogramm. Remote Sens. Spat. Inf. Sci..

[54] Wierzbicki D., Matuk O., Bielecka E. (2021). Polish Cadastre Modernization with Remotely Extracted Buildings from High-Resolution Aerial Orthoimagery and Airborne LiDAR. Remote Sens..

[55] Rottensteiner F., Trinder J., Clode S., Kubik K. (2005). Using the Dempster–Shafer method for the fusion of LIDAR data and multispectral images for building detection. Inf. Fusion.

[56] Sohn G., Dowman I. (2007). Data fusion of high-resolution satellite imagery and LIDAR data for automatic building extraction. ISPRS J. Photogramm. Remote Sens..

[57] Khoshboresh-Masouleh M., Alidoost F., Hossein A. (2020). Multiscale building segmentation based on deep learning for remote sensing RGB images from different sensors. J. Appl. Remote Sens..

[58] Kocur-Bera K., Stachelek M. (2019). Geo-Analysis of Compatibility Determinants for Data in the Land and Property Register (LPR). Geosciences.

[59] Hanus P., Benduch P., Pęska-Siwik A. (2017). Budynek na mapie ewidencyjnej, kontur budynku i bloki budynku. Przegląd Geod..

[60] Buśko M. Modernization of the Register of Land and Buildings with Reference to Entering Buildings into the Real Estate Cadastre in Poland. Proceedings of the International Conference on Environmental Engineering.

[61] Lalak M., Wierzbicki D. (2022). Methodology of Detection and Classification of Selected Aviation Obstacles Based on UAV Dense Image Matching. IEEE J. Sel. Top. Appl. Earth Obs. Remote Sens..

